# Education of Adolescents in the Prevention of HIV/AIDS in the Czech Republic

**DOI:** 10.3390/ijerph18116148

**Published:** 2021-06-07

**Authors:** Petra Macounová, Hana Tomášková, Anna Šnajdrová, Markéta Stanovská, Martina Polochová, Ivan Tomášek, Rastislav Maďar

**Affiliations:** 1 Department of Epidemiology and Public Health, Faculty of Medicine, University of Ostrava, 709 00 Ostrava, Czech Republic; petra.macounova@osu.cz (P.M.); marketa.stanovska@osu.cz (M.S.); martina.polochova@osu.cz (M.P.); rastislav.madar@osu.cz (R.M.); 2 Public Health Institute Ostrava, 702 00 Ostrava, Czech Republic; anna.snajdrova@szu.cz (A.Š.); ivan.tomasek@zuova.cz (I.T.)

**Keywords:** HIV, health promotion, health education, sexually transmitted diseases, primary prevention, preventive programs

## Abstract

The number of HIV-positive people in the Czech Republic has trebled over the last decade. An educational programme aimed at the prevention of HIV and STDs in adolescents between 13 and 19 years of age is underway in the Moravian-Silesian Region in the Czech Republic. All schools in the region have been offered the chance to participate in the programme (on a voluntary basis). The programme consists of an educational lecture and a game with interactive elements; the students perform various tasks in groups. An anonymous paired questionnaire (input/output) was used to assess the efficacy of the programme. In order to evaluate the programme efficacy, descriptive statistics, non-parametric Wilcoxon paired test at the level of significance of 5%, and a model of logistic regression for a dichotomous variable were used. A total of 1210 students took part in the programme. The students’ knowledge improved, on average, by 15.5%. The highest efficacy was observed in the age group of 13–14-year-olds, where the improvement reached 17.9%. The educational programme was effective in raising awareness about STDs. Due to the observed increase in knowledge especially among the youngest participants (13–14 years old), we recommend aiming further interventions at the youngest age groups.

## 1. Introduction

Although many years have passed since the discovery of the HIV/AIDS disease, it still represents a significant global problem [1,2]. At the end of 2018, approximately 37.9 million people worldwide were infected with HIV. Despite the worldwide availability of testing, 21% of all HIV+ patients are not aware of their condition [3]. The Czech Republic counts among countries with a relatively low incidence of this disease; nevertheless, it belongs, at the same time, to the list of countries with a rapid increase in the number of new cases of HIV infection over the last few years [4,5]. The number of new cases has grown approximately three times from 1186 in 2008 to 3368 in 2018 [6]. HIV infection remains, despite significant advancements in treatment, an incurable disease, which is not preventable by vaccination. Hence, primary prevention of HIV infection plays an important role and, at the same time, remains the most effective and most economical preventive measure. Raising awareness among the youth, general public, and specific populations with a high risk of infection using training and education about sexually transmitted diseases (hereinafter referred to as STDs), including HIV/AIDS, is the cornerstone of primary prevention measures [7,8,9,10,11].

In addition to the education of pupils and students of primary and secondary schools, educational events, peer programmes, and lectures with an HIV+ lecturer, primary HIV/AIDS prevention also includes services of AIDS counselling, contact centres, and exchange of used injection needles as a part of the harm reduction programme [12,13,14,15]. In the area of raising awareness and education, it is necessary to apply preventive measures especially towards the youth (in particular, in the areas of prevention of disease transmission, risk behaviour, and promotion of safe sex); it is also important to motivate and encourage responsibility for one’s acts, using condoms, and healthy behaviour [16].

The Public Health Institute in Ostrava has implemented educational events for schools, including the interactive programme Playfully about STDs (PaSTDs), the aim of which is to raise awareness about the possibilities of preventing the transmission of the HIV virus and other sexually transmitted diseases in young people, and to support the development of responsible and desirable behaviour in the area of STD prevention, including HIV, and, thus, to reduce risky behaviour [17,18].

The aim of the study is to present students’ knowledge of HIV/AIDS and the effectiveness of the PaSTDs educational programme in raising awareness and knowledge regarding prevention of HIV and STDs in adolescents. The basic hypothesis is that this programme represents an effective tool of primary prevention and that after its completion, the participants’ knowledge about the problems of HIV and STDs increases.

## 2. Materials and Methods

### 2.1. Design

An educational programme aimed at the prevention of HIV and STDs in adolescents between 13 and 19 years of age is underway in the Moravian-Silesian Region in the Czech Republic. All grammar and secondary schools (a total of 457 schools) in the region have been offered the possibility to take part in the programme; their participation was voluntary. Over 2 years (2018–2019), students from 31 schools in the Moravian-Silesian Region, Czech Republic, participated in the programme. A total of 1264 students aged 13 to 19 present on the day of the educational programme at the school participated in the programme.

The programme consists of an educational lecture (two lessons) and a game with interactive elements, during which the pupils from each class are divided into 4 groups and perform 8 tasks with various educational tools (magnetic board and magnetic cards, samples of contraception methods, models of penis for training of condom handling), for which they receive points in the form of special “bouncing balls” [17]. A more detailed description of the course of the programme can be found in Appendix A.

### 2.2. Measures

An anonymous paired questionnaire (input/output) was used to assess the efficacy of the programme. The input questionnaire, with a unique ID, was distributed among the students prior to commencement of the educational programme; this questionnaire served as the baseline evaluation of participants’ knowledge about the problems of HIV before education. The questionnaire contains 12 questions, beginning with the data about the participant—sex and age. The student subsequently answers 10 test questions on sexually transmitted diseases, choosing from multiple-choice options (the answer “I don’t know” is always one of the options). After education, the students filled in the output questionnaire with the same ID and with questions identical to those in the input questionnaire, supplemented with the possibility to evaluate the programme on a scale from 1 to 5 and to add an open comment.

The questionnaire used in this study was based on the one published in our previous pilot study about the educational programme [17]. In addition, two questions (8, 9, see Appendix A) in the questionnaire were changed during data collection.

### 2.3. Data and Variables

A total of 1264 students participated in the preventive programme; 54 students were excluded from the analysed sample due to incomplete/incorrect filling of the questionnaires (e.g., ticking multiple options in single-choice questions or using ambiguous indicators). The questionnaire was also excluded from analysis if the pair (input and output part of the questionnaire) was incomplete. The final sample consists of 1210 students. 

Data about sex, age (age groups 13–14 years, 15–16 years, 17 and older) and the type of school—a grammar school (GS), a secondary school (SS), or a lower secondary school (LSS)—were collected. Test questions were awarded one point for each correct answer. The maximum was 22 points. The total score was expressed as a percentage of the maximum. The two questions that were changed over the course of data collection (questions 8, 9—Appendix A) were excluded from the total score. The total score was calculated separately for the input and output questionnaire. Students’ evaluation of the programme quality was on a scale from 1 (the best) to 5 (the worst).

EpiData Software was used for the electronisation of the data from paper questionnaires.

### 2.4. Data Analysis

Descriptive statistics was used for the basic presentation of the results. The changes in the proportions of correct answers in each of the test questions were evaluated via the McNemar test. The non-parametric Wilcoxon paired test was used to test the changes in the total score (before and after education). The power of the mean difference between total scores before and after education was calculated based on a one-sample mean test for a one-sided alternative hypothesis (Ha: mean difference > 0). The comparison of the total score by sex, age, and school type was performed using Mann–Whitney and Kruskal–Wallis tests. A model of logistic regression for dichotomous variables was used to evaluate the most significant factors affecting the participants’ knowledge. Low knowledge was defined as a total score of less than 70%. The basic (reference) categories were set as: men (for the sex variable), the youngest students (13–14 years; age variable), and grammar school (the type of school). The results were expressed as odds ratio (OR) with standard error (SE) and 95% confidence interval (CI). Statistical tests were evaluated at the 5% significance level. The Stata software, version 14, was used to analyse the data.

### 2.5. Ethical Considerations

The study was approved by the Ethics Committee of the Faculty of Medicine of the University of Ostrava on the basis of the EC Consent Opinion No. 04/2020.

## 3. Results

The age span of participants was 13–19 years, with a mean age of 14.6 years; 4 participants did not state their age. The representation of boys and girls in the group was almost even, with a slightly higher number of boys. Thirteen participants did not fill in the information about their sex. The analysed group consisted of a total of 1210 pupils, with 987 pupils from lower secondary schools (LSS), 137 pupils from secondary schools (SS), and the remaining 86 students from grammar schools (GS) (Table 1).

### 3.1. Questionnaire Results

The principal question in the questionnaire related to the awareness of pupils about what HIV is; only 48.7% of pupils selected the correct answer in the questionnaire. The number of correct answers increased to 84.7% after the completion of the course, clearly indicating an increase in the correct answers by more than 36.0%. Almost 80% of participants responded correctly to the question “What disease is caused by the HIV virus?” before the programme. Only less than 10% of the participants selected an incorrect disease; however, it is important to note that a relatively high number of students, 10.8%, chose the option “I don’t know” (see enclosed Table 2) at that stage.

The knowledge of pupils in the area of transmission and spreading of the HIV virus was relatively satisfactory even before education. A high number of respondents correctly answered the question regarding the transmission of HIV through sexual intercourse; the answers did not differ significantly in this area between the input and output questionnaires (more than 95% in both cases). The lowest degree of knowledge was recorded regarding HIV transfer from a positive mother to her child, with only 66.7% of correct answers before and 89.9% after education (i.e., with a significant increase of more than 23%). A significant increase in knowledge after education was also observed in the questions concerning the means by which HIV cannot be transmitted, i.e., common kissing or insect bites (see Table 2).

Unsatisfactory results prior to education were observed in several very important HIV-related issues—namely, whether there is a vaccination against HIV (only 56.5% of correct answers before the educational programme significantly increasing to 83.1% after the programme). A limited number of respondents believed that an effective vaccination against HIV exists (23%), and a relatively higher number of students selected the option “I don’t know” (20%). Similar results were also seen where the knowledge of hormonal contraception in the sense of HIV prevention was concerned. The success rate of answers was also very low in this question, reaching only 62.0% before the programme and increasing to 90.2% after education. 

Two questions of a predominantly informative character (the town with the highest number of HIV-positive individuals within the Czech Republic and the trend in the number of HIV-positive patients) were answered by 949 students and, subsequently, substituted with more important questions that can be used internationally. These new questions were answered by 261 students. The question about the time interval for HIV testing after a risky situation needed to obtain a valid result was correctly answered by 16.5% of students prior to education and by 75.4% of students after the programme. The question of whether a condom is considered an important protection means against HIV infection was correctly answered by 80.0% of respondents before, and 94.6% after the education.

The median total point score before education was 17 points (range 6–22 points, average % score 74.9%); after education, the total point score increased to 21 points (range 8–22 points, average total % score 89.4%). The mean difference between the total % scores after and before education was 15.5%. The difference between total % scores was statistically significant, and the power of the test was high (*p* < 0.001; power = 100) (see Table 3).

### 3.2. What Factors Affect Knowledge?

Factors significantly influencing the participants’ knowledge about HIV/AIDS were also analysed. Table 4 presents the results of comparing the scores before and after the programme divided by sex, age, and type of school. Significant differences were observed prior to education, with the exception of male-to-female comparison. After education, girls’ knowledge was higher (average success rate 91.4%) than boys’ (87.8%). The lowest mean values before education were observed in lower secondary school pupils (72.6%); after education, students at secondary schools manifested the lowest scores (87.7%). The level of knowledge before education increased with age before education, while after it, the worst results were recorded in the oldest students. Hence, the highest effect of education was observed in the age group of 13–14-year-olds, with an improvement of the questionnaire results by almost 18% (from 71.1% to 89.0%). In comparison, the average improvement in the 17+ group, where the knowledge level was the highest already at the beginning, was only 8.1%; nevertheless, the improvement was still statistically significant.

### 3.3. Which of These Factors Are Significant?

**Figure 1:**
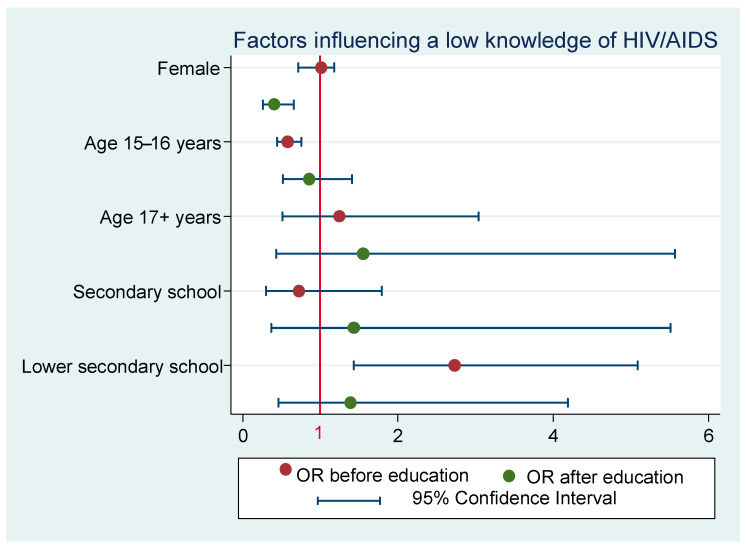
Results of models monitoring the influence of gender, age, and type of schools on the low knowledge of HIV/AIDS (1: Total % score < 70). OR—odds ratio; baseline—male, age 13–14 years, grammar school. Model (before education) fully adjusted: *p* = 0.025; model (after education) fully adjusted: *p* < 0.001.

A low level of knowledge (a total score < 70%) was identified in 36% of students before education and in 8% of students after education. Statistically significant factors—age and type of school—were identified in the fully adjusted model before education. The age group of 15–16-year-olds already showed significantly higher knowledge before education than the group of 13–14-year-olds (OR = 0.58; SE = 0.81; 95% CI: 0.44–0.76). Lower secondary school pupils achieved statistically significantly worse point scores when compared with grammar school students (OR = 2.73; SE = 0.87; 95% CI: 1.46–5.09). After education, the differences according to age and type of school were no longer significant (see Figure 1); however, a significantly greater improvement of the knowledge level was observed in girls than in boys (OR = 0.41; SE = 0.10; 95% CI: 0.26–0.66).

In the final part of the output questionnaire, the participants evaluated the programme on a scale from 1 (best) to 5 (worst); the average evaluation was 1.23, which means that the students rated the programme highly positively; however, the evaluation decreased with age.

## 4. Discussion

In addition to the PaSTDs programme, there is also another project in the Czech Republic educating young people and forming their attitudes in the field of HIV/AIDS prevention. This project is organised by the National Institute of Public Health under the name “Playing Against AIDS”. In this project, all information regarding HIV is presented to students only by means of oral presentations using available tools, and the programme does not include any educational “hands-on” presentation. Participants work in five individual groups, and the significant gaming character is missing. Each group of participants has their own lecturer, and the programme is more personally demanding when compared with the PaSTDs project, requiring only one lecturer for the whole intervention. The PaSTDs project uses an educational game to present the subject and input and output questionnaires to determine the effectiveness of the programme. Nevertheless, based on available sources, both programmes are of high quality, effective, and positively evaluated [19]. The educational information of PaSTDs was evaluated positively by the students; their interest in the HIV issue manifested especially through the increase in awareness about this problem by more than 15%.

The knowledge level increased in a vast majority of the 1210 participants; therefore, we can consider the programme effective. The programme was most effective in the youngest age group of 13–14-year-olds, where the improvement in the knowledge level was almost 18%. This is most probably caused by the fact that the youngest participants do not have much information and experience yet (as corroborated by their lowest initial result); this result may have also been due to the gaming/competitive character of the programme, which seems to be more attractive to younger participants. The average improvement observed in the 15–16-year-olds was 13.66%. Compared to that, the oldest age group of 17–19-year-olds achieved an average improvement of a mere 8.1%, which is most likely due to the already high level of knowledge the students had before completing the programme. The highest increase in the knowledge level was observed, in particular, in the most important questions, such as what HIV is, whether there is a vaccination against HIV, or whether hormonal contraception provides protection against HIV infection. It is also possible to evaluate the knowledge with respect to sex, which shows that compared to boys, girls not only performed better before the education, but the increase in their knowledge was also higher.

Although most educational programmes dealing with the problem of HIV/AIDS infection and other STDs are primarily aimed at children and youth, these interventions are also important for other population groups. For example, a positive influence of the intervention has been previously confirmed among drug users [20]. Educational programmes and their implementation play an important role in increasing knowledge and awareness about HIV/AIDS. Although HIV/AIDS infection is not going to disappear completely as a result of these programmes, they may help to reduce the number of new cases of these infections; it is, therefore, appropriate to continue with such programmes, to develop them further and adjust them to the ever-changing requirements [16].

The positive effect of educational programmes is also supported by a meta-analysis summarising 83 studies from various countries (developed as well as developing), aiming at sex education and the prevention of HIV and other sexually transmitted diseases. The results of the analysis show a positive impact of educational programmes in this area on the behaviour of the adolescent population [21].

Current findings show that these interventions are effective worldwide. According to a systematic review examining the effect of interventions on the prevention of unwanted pregnancy from 2016, multiple interventions combining education and promotion of contraceptive methods significantly reduce the risk of unintentional pregnancy in adolescents. The review further revealed that educational interventions alone will most probably not result in postponing the beginning of sexual life in adolescents; however, the frequency of condom use increases significantly when compared to individuals who have not been through such an intervention [22]. This is also why our programme emphasises the importance of using a condom as well and why it also contains a practical demonstration of its use. Another part of the programme is a demonstration of other contraceptive methods, including hormonal contraception, for which it is always clearly emphasised that they provide protection only against unwanted pregnancy but not against HIV and STDs.

This may also be the reason preventive programmes encouraging only sexual abstinence have been lately criticised as ineffective and, in addition, principally not promoting the strategy of safer sex in the sense of STDs prevention (use of condoms) and protection against unwanted pregnancy (use of condoms and other methods of contraception). This criticism is also supported by the findings of a previous study on the effectiveness of interventions aimed solely at sexual abstinence as prevention of HIV infection, which concluded that such programmes are ineffective. The results did not show that these programmes reduce the risk of HIV infection; moreover, these programmes failed to influence the incidence of unprotected vaginal sex, frequency of vaginal sex, number of partners, sexual initiation, or use of condoms [23].

Thus, it is clearly apparent that it is more effective to lead adolescents towards responsible sexual behaviour than trying to force them into sexual abstinence as the only means of prevention. This is also indicated by the meta-analysis published in 2011, which concentrated directly on interventions intended to reduce the sexual risks of HIV infection in adolescents. A total of 98 interventions from 67 studies were included in this meta-analysis; these were completed in total by 51,240 adolescents aged 11–19 years. The findings show that the incidence of sexually transmitted diseases decreased among those who completed the education when compared with controls; additional results included more frequent use of condoms, delaying sexual intercourse or decreasing its frequency, and upgrading the skills required for applying the principles of safe sex and obtaining prophylactic protection [24].

Another study performed in Tanzania investigated the degree to which education based on games and gamification (i.e., using gaming elements and principles in education) could improve the education of adolescent students on sexual health when compared with the traditional style of education. A total of 120 students from secondary schools took part in the programme. The average score after completing the test for education based on games and gamification, which reached 79% in both these educational methods, was significantly higher than in the control group, where it reached only 51%. This study suggests that the investigated innovative approaches to education may improve education on sexual health in the adolescent population. These methods may potentially contribute, in particular, to improving behaviour in the area of sexual health and increasing the knowledge of adolescents, especially in contexts where discussions concerning sexual problems present a taboo [25]. This is also why involving gaming elements is used in our programme—the intention is to increase the attractiveness of the programme and to eliminate possible shame and shyness in the participants.

Should we consider the generalisability of the results, it is likely that a similar effect in the form of improving the participants’ knowledge could also be observed in the target age group of 13–19-year-olds in other regions of the Czech Republic. Possible use of the programme in other countries clearly depends on the contents of the educational programmes at schools in individual countries, especially the inclusion of subjects similar to the Health Education subject taught in the Czech Republic. However, this subject is taught at schools for one year only and, considering the answers to some questions in the questionnaire, it is possible to claim that the problems of HIV and STDs are not given sufficient attention in the course of standard school education. Similar findings were also observed in the questionnaire survey performed by the National Institute of Public Health in 2015, which revealed that adolescents are not sufficiently educated in this area and that more attention needs to be paid to these topics in the future [26]. According to another study from 2014, school educational programmes about HIV/AIDs are an important and effective means of influencing the knowledge, attitudes, and behaviour of young people regarding health issues related to sex. The study showed that educational programmes about HIV/AIDS at schools may effectively reduce the risky behaviour of young people. The authors also propose that education in the area of HIV/AIDS prevention should ideally start before puberty, or, at the latest, before the first sexual intercourse [15]. We also tend to support this conclusion, considering the highest effectiveness observed in the youngest age group of our programme.

The PaSTDs programme should be based on the principle of double transmission of information—the first time during the educational lecture, and the second time during the game. In addition, the effectiveness is also supported by the initial questionnaire before the programme as students who did not know answers to individual test questions were more likely to extract the answers from the given lecture.

Limitations: The greatest limitation of this programme may be the fact that it represents a one-off intervention only and there is no further monitoring, for example, of the length of knowledge persistence, or of possible effects on the future risky behaviour of the participants. However, these drawbacks are mainly caused by a lack of financial means invested in the area of prevention and, in our case, also the limited staffing of the programme. The first and greatest obstacle in the implementation of the programme was the lack of interest in the programme at some schools. We propose performing a repeated intervention in the already educated pupils in order to discover whether the knowledge persists on a long-term basis. The programme is currently interrupted due to the complete closure of schools in the Czech Republic caused by the coronavirus pandemic; nevertheless, we expect it to continue when the situation permits.

## 5. Conclusions

Taking into consideration the presented results, it is possible to conclude that the programme leads to increased knowledge in the participants and, as such, can be considered an effective tool of primary prevention in the sense of increasing awareness about the problem of HIV and STDs by 15.5% on average (from 74.0% to 89.5%).

The programme was observed to be more effective in lower age categories (the score in the age group of 13–14-year-olds improved by 17.92%). Evaluation of knowledge according to sex (M/F) shows that girls achieved a higher success rate of answers before education and, at the same time, manifested a higher increase in knowledge after education than boys.

The participating students showed great sympathy with the educational programme; their interest in this problem corresponds to a significant increase in knowledge observed especially among the youngest participants. This is also why we propose to focus further interventions, in particular, on this youngest age group of 13–14-year-olds.

## Figures and Tables

**Table 1:** Description of the sample (*n* = 1210).

Variable	Category	Number	%
Gender	Male	609	50.9
	Female	588	49.1
School	Grammar school	86	7.1
	Secondary school	137	11.3
	Lower secondary school	987	81.6
Age (years)	13–14	648	53.7
	15–16	466	38.7
	17+	92	7.6

**Table 2:** Selected questions and correct answers before and after education (*n* = 1210).

Question/Statement (the Correct Answer)	% Correct Answers		Change	*p*-Value *
	Before Education	After Education		
1. What is HIV?	48.7%	84.7%	**36.0%**	<0.001
2. What disease is caused by HIV?	77.1%	87.4%	**10.3%**	<0.001
3. HIV modes of transmission:				
Insect sting or bite (NT)	91.2%	98.4%	**7.1%**	<0.001
Kissing (NT)	88.2%	97.1%	**8.9%**	<0.001
From HIV+ mother to child (T)	66.7%	89.9%	**23.2%**	<0.001
Sharing a toilet (NT)	92.6%	95.0%	**2.4%**	0.008
Sharing needles and syringes (T)	71.9%	89.8%	**17.9%**	<0.001
Blood (transfusion) (T)	67.9%	80.3%	**12.4%**	<0.001
Hug, handshake (NT)	98.9%	99.3%	**0.3%**	0.371
Sneezing and coughing (NT)	89.9%	97.5%	**7.6%**	<0.001
Sexual transmission (T)	96.3%	96.9%	**0.6%**	0.354
4. HIV vaccination exists (NT)	56.5%	83.1%	**26.5%**	<0.001
5. Hormonal contraception acts as HIV prevention (NT)	61.9%	90.2%	**28.2%**	<0.001
6. Complete cure of HIV is possible (NT)	72.2%	92.5%	**20.3%**	<0.001
7. HIV in Czechia is more common among men	47.9%	84.4%	**36.5%**	<0.001
8. (original ^1^) Trend in the number of HIV+ in the Czech Republic over the last 10 years: rising	60.4%	92.5%	**32.1%**	<0.001
9. (original ^1^) City with the largest number of HIV+ in Czech Republic: Prague	33.1%	84.2%	**51.1%**	<0.001
10. (new ^2^) Condom as a protection against HIV: is significant	80.0%	94.6%	**14.6%**	<0.001
11. (new ^2^) Period to valid testing after a risky contact (2 to 3 months)	16.5%	75.4%	**58.9%**	<0.001

* McNemar test; NT = not true; T = true. ^1^ These questions were not included in the total score (*n* = 949); ^2^ These questions were not included in the total score (*n* = 261). The bold in the table is a significant output that should be made visible.

**Table 3:** Total scores before and after education (*n* = 1210).

Total Score	Period	Median	Mean	SD	*p*-Value ^2^/Power ^3^
Points	Before education	17	16.3	3.17	<0.001
	After education	21	19.7	2.69	
**%**	Before education	73.9	74.9	14.42	<0.001
	After education	95.5	89.4	12.23	
Difference ^1^		13.4	15.5	14.50	100%

^1^ Difference = total % score after education—total % score after education; ^2^ Wilcoxon paired test. ^3^ The power for the mean difference was calculated based on a one-sample mean test for one side alternative hypothesis (Ha: mean difference > 0).

**Table 4:** Average values of total scores (%) before and after education by sex, age, and type of school.

Variable	Category	*n*	Total % Score Before Education			Total % Score After Education			*p*-Value ^3^
			Median	Mean	SD	Median	Mean	SD	
Gender	Male	609	75	**73.1**	14.27	91.7	**87.8**	13.23	<0.001
	Female	588	75	**74.9**	13.68	95.7	**91.4**	9.99	<0.001
*p*-value ^1^			0.0351			0.001			
School	GS *	86	85.4	**81.1**	14.37	100	**95.2**	8.78	<0.001
	SS **	137	83.3	**79.7**	13.12	91.7	**87.7**	14.34	<0.001
	LSS ***	987	75	**72.6**	13.80	91.7	**89.2**	11.74	<0.001
*p*-value ^2^			0.001			0.001			
Age (years)	13–14	648	70.8	**71.1**	13.94	91.7	**89.0**	11.54	<0.001
	15–16	466	79.2	**77.1**	13.29	95.8	**90.7**	12.03	<0.001
	17+	92	83.3	**78.4**	14.52	91.7	**86.5**	14.24	<0.001
*p*-value ^1^			0.001			<0.001			

^1^ Mann–Whitney test; ^2^ Kruskal–Wallis; ^3^ Wilcoxon paired test; * GS—grammar school; ** SS—secondary school; *** lower secondary school. The bold in the table is a significant output that should be made visible.

## Data Availability

The data presented in this study are available on request from the corresponding author. The data are not publicly available due to their quantum.

## Appendix A

The programme description: The programme was implemented directly in individual schools and classes, where the data were also collected.

The input questionnaire, with a unique ID, is distributed among the students prior to the implementation of the educational programme; the aim of this questionnaire is to reveal knowledge on HIV-related issues before education. The questionnaire contains 12 questions, the first 2 of which identify basic data regarding the respondent—sex and age. The student subsequently answers test questions pertaining to sexually transmitted diseases, choosing from multiple-choice options, which are always supplemented with the answer “I do not know”. Two questions in the questionnaire were changed during data collection.

The list of the test questions and their maximum scoring points are detailed in the table below:

**Table A1:** The list of the test questions.

1. What is HIV? *(1 point)*
2. Which disease is caused by HIV? *(1 point)*
3. HIV modes of transmission: (10 points, 1 for each correct answer)
Insect stings or bite; Kissing; From HIV+ mother to child; Sharing a toilet; Sharing needles and syringes; Blood (transfusion); Hug; Handshake; Sneezing and coughing; Sexual transmission.
4. HIV vaccination does/does not exist. *(1 point)*
5. Can hormonal contraception prevent HIV? *(1 point)*
6. Is HIV completely curable? *(1 point)*
7. In which sex does HIV occur more frequently in the Czech Republic?*(1 point)*
8. (old) What is a trend in the number of HIV+ in the Czech Republic over the last 10 years? *(0 points)*
9. (old) Which city has the largest number of HIV+ individuals in the Czech Republic? *(0 points)*
10. (new) Does a condom provide protection from HIV? *(0 points)*
11. (new) What is the time interval from a risk contact, after which HIV test is valid? *(0 points)*
12. Which of the diseases are not STDs? *(6 points)* Smallpox; Syphilis; Gonorrhoea; Tetanus; AIDS; Measles.

After filling in the input questionnaire, the students complete the programme itself, which consists of an educational lecture and a game, and takes 2 lessons, i.e., 90 min. The educational lecture is a presentation bringing information about HIV/AIDS and STDs, which helps the students with solving tasks during the following educational games. The lecture starts with an initial question of the lecturer in the form of brainstorming, where the lecturer asks the students about their first thoughts upon hearing the term “sexually transmitted diseases”.

The game consists of 8 tasks with various educational tools (magnetic board and magnetic cards, samples of contraception methods, models of penis for training of condom handling), supplemented with a discussion, by means of which the students learn about this topic. In the beginning, the lecturer gives students information about the course of the game; it is important to put emphasis on the fact that the students should bear in mind that the game is teamwork-oriented, not single-player with a competitive character.

Students are then divided into equally sized groups, ideally with mixed sexes. After each accomplished game, the winning group is rewarded with so-called bouncing balls. The primary aim of using the bouncing balls is to increase the motivation of students to be active during the game; it is also possible to take the bouncing balls back away from the students for disruptions during the game, and to award bonus bouncing balls; nevertheless, the lecturer informs the students that the reward is purely symbolic, and winning the game is not the most important thing. In cases of extreme disruption of the programme and non-cooperation (especially in students with behavioural disorders), such students may be expelled from further participation in the programme.

The students then receive the output questionnaire, which serves to determine the effectiveness of the programme. It contains questions identical to those in the input questionnaire, supplemented with the possibility to evaluate the programme on a scale from 1 to 5 and to add an open comment. After completion of the programme, each student takes home educational materials and a condom.
